# Two Saporin-Containing Immunotoxins Specific for CD20 and CD22 Show Different Behavior in Killing Lymphoma Cells

**DOI:** 10.3390/toxins9060182

**Published:** 2017-05-30

**Authors:** Letizia Polito, Daniele Mercatelli, Massimo Bortolotti, Stefania Maiello, Alice Djemil, Maria Giulia Battelli, Andrea Bolognesi

**Affiliations:** Department of Experimental, Diagnostic and Specialty Medicine—DIMES, General Pathology Section, Alma Mater Studiorum—University of Bologna, Via S. Giacomo 14, 40126 Bologna, Italy; danielemercatelli@gmail.com (D.M.); massimo.bortolotti2@unibo.it (M.B.); stefania.maiello2@unibo.it (S.M.); alice.djemil2@unibo.it (A.D.); mariagiulia.battelli@unibo.it (M.G.B.); andrea.bolognesi@unibo.it (A.B.)

**Keywords:** B-lymphoma, CD20, CD22, immunotherapy, immunotoxin, ribosome-inactivating proteins, saporin-S6

## Abstract

Immunotoxins (ITs) are hybrid proteins combining the binding specificity of antibodies with the cytocidal properties of toxins. They represent a promising approach to lymphoma therapy. The cytotoxicity of two immunotoxins obtained by chemical conjugation of the plant toxin saporin-S6 with the anti-CD20 chimeric antibody rituximab and the anti-CD22 murine antibody OM124 were evaluated on the CD20-/CD22-positive cell line Raji. Both ITs showed strong cytotoxicity for Raji cells, but the anti-CD22 IT was two logs more efficient in killing, probably because of its faster internalization. The anti-CD22 IT gave slower but greater caspase activation than the anti-CD20 IT. The cytotoxic effect of both immunotoxins can be partially prevented by either the pan-caspase inhibitor Z-VAD or the necroptosis inhibitor necrostatin-1. Oxidative stress seems to be involved in the cell killing activity of anti-CD20 IT, as demonstrated by the protective role of the H_2_O_2_ scavenger catalase, but not in that of anti-CD22 IT. Moreover, the IT toxicity can be augmented by the contemporary administration of other chemotherapeutic drugs, such as PS-341, MG-132, and fludarabine. These results contribute to the understanding of the immunotoxin mechanism of action that is required for their clinical use, either alone or in combination with other drugs.

## 1. Introduction

Non-Hodgkin’s lymphomas (NHLs) are a group of heterogeneous hematological malignancies with a wide range of aggressiveness. The majority of NHLs involve B-cells. Although NHLs are quite curable, approximately 50% of NHL patients either relapse or become refractory to conventional therapy. For these patients with poor outcomes, it is mandatory to find new therapeutic strategies, such as monoclonal antibody (mAb)-based immunotherapy. Several receptors are widely expressed on the surface of B-cell lymphomas. Two of them, CD20 and CD22, are expressed at high levels on normal mature B-cells and on a vast proportion of B-lymphoma cells but are absent from other normal tissues and hematopoietic stem cells. Efforts have been made toward developing mAbs that would specifically bind to CD20/CD22 as therapeutic agents for B-cell NHLs. The in vivo administration of these mAbs confirmed their high-affinity binding to B-lymphoma cells, while other hematological cells, such as T cells and stem cells, were not recognized. The positive outcome of clinical use of the anti-CD20 mAb rituximab represents a good example of the worldwide interest in anti-B-cell immunotherapy [1,2].

The cytotoxic effects of mAbs can be enhanced by their conjugation with drugs, radioisotopes, or toxic enzymes. The latter type of conjugates is classified as immunotoxins (ITs), which are chimeric proteins with specific cytotoxic effects [3,4]. Both plant and bacterial toxins can be utilized for the preparation of ITs, which are mostly designed for cancer immunotherapy and are particularly suitable for eliminating residual disease after chemo- or radiotherapy. To date, ITs have achieved excellent results in several different models in preclinical settings [5,6,7] and clinical trials [6,8,9]. The most promising results have been reported in the treatment of hematological malignancies [5,9]. 

Among plant toxins, ribosome-inactivating proteins (RIPs) have been widely used to produce ITs [10,11]. RIPs are largely distributed in the plant kingdom [12], and many plants producing RIPs have been used for centuries in traditional medicine [13]. RIPs are mainly divided into type 1, consisting of a single-chain enzymatic protein, and type 2, consisting of an A chain with the same enzymatic activity linked to a B chain with lectin properties [14]. The A chain cleaves one or more adenine molecules from ribosomal RNA, thus irreversibly damaging the ribosomes. The literature about type 1 RIP saporin is very abundant, comprising more than a thousand studies. Saporin-S6 has been well characterized, and because of its potency and stability, it has been largely employed in the production of conjugates and ITs for various purposes [9,15,16,17,18,19,20] and sometimes has been tested in clinical trials [9,11,21]. Saporin-S6, as many other type 1 and type 2 RIPs, can remove adenine from various biochemical targets in vitro, including ribosomal RNA, DNA, poly(A), and poly-ADP-ribosylated proteins [22,23,24]. Most likely, saporin-S6 is able to deadenylate different substrates also in vivo, and, as a consequence, once internalized in target cells, it can trigger several death pathways, including apoptosis, necroptosis, and to a lesser extent, necrosis [25]. This multipathway killing could also depend on RIP distribution inside the cell. Indeed, saporin-S6 has been shown to have a different intracellular routing path compared to other RIPs, such as ricin [26]. The accumulation of saporin-S6 in the endoplasmic reticulum and perinuclear cisternae has previously been reported [27] and could be in agreement with the unfolded protein response (UPR) evidenced for other RIPs [28]. Moreover, molecules of saporin-S6 were detected inside the nuclei of intoxicated cells together with DNA strand break formation, thus suggesting direct saporin-S6 action on nuclear DNA [27]. 

In previous studies, we demonstrated that the anti-tumor efficacy of both the anti-CD20 mAb rituximab and the anti-CD22 mAb OM124 can be strongly augmented by their conjugation to RIPs [15,16]. In this research, we compared the cytotoxic effects of two immunoconjugates obtained by the chemical linking of saporin-S6 with the two mAbs, rituximab and OM124, on the CD20-/CD22-positive Raji cell line. CD20 and CD22 were chosen as target antigens in our experiments because of their large expression on B-cell malignancies [1,2]. The cell death pathways involved were investigated both with direct methods and indirectly by using inhibitors of specific routes, such as the pan-caspase inhibitor Z-VAD, the necroptosis inhibitor necrostatin-1 (Nec-1) and the H_2_O_2_ scavenger catalase. Moreover, we explored the possibility of specifically augmenting the cytotoxic activity of the ITs with two proteasome inhibitors, PS-341 and MG-132, and with the chemotherapeutic drug fludarabine with the aim of increasing their efficiency in killing target cells.

## 2. Results

### 2.1. Immunotoxin Purification and Characterization

**Figure 1:**
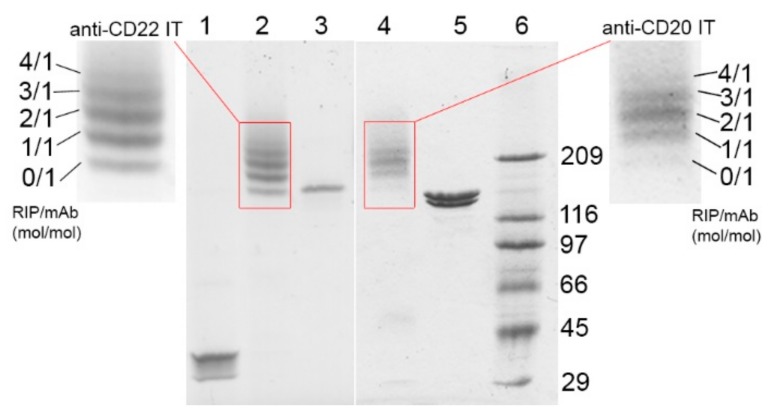
Analysis of rituximab/saporin-S6 (anti-CD20 IT) and OM124/saporin-S6 (anti-CD22 IT) immunotoxins by SDS–PAGE under non-reducing conditions. The gel was stained with Coomassie blue and subjected to densitometric analysis to establish the ribosome-inactivating protein (RIP)/mAb molar ratio. Lane 1: unconjugated saporin-S6, lane 2: anti-CD22 IT, lane 3: unconjugated OM124, lane 4: anti-CD20 IT, lane 5: unconjugated rituximab, lane 6: Mw standards expressed in kDa.

**Figure 2:**
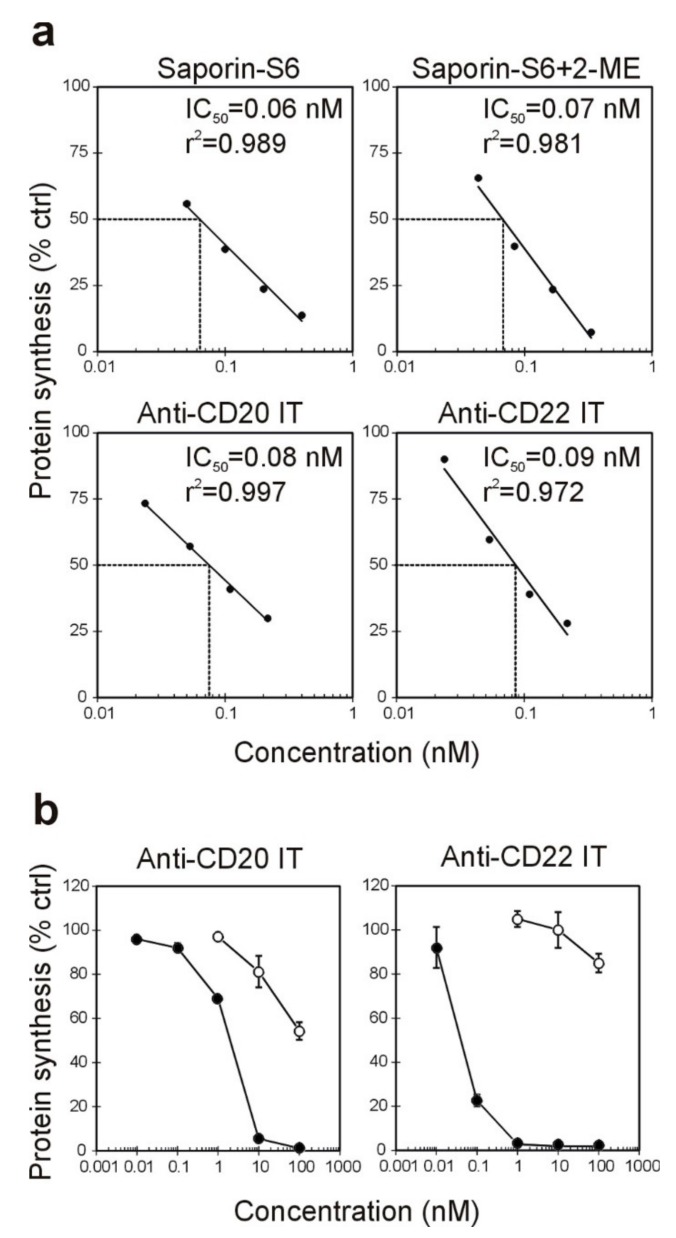
(**a**) The protein synthesis inhibitory activity of the two immunotoxins (ITs) and free RIP was evaluated using a cell-free rabbit reticulocyte lysate system. The IT rituximab/saporin-S6 (anti-CD20 IT) or OM124/saporin-S6 (anti-CD22 IT) and saporin-S6 were reduced with 20 mM 2-mercaptoethanol (2-ME) for 30 min at 37 °C and compared to native saporin-S6. The IC_50_ is the concentration of free or conjugated RIP causing 50% protein synthesis inhibition and was calculated by linear regression analysis. (**b**) Protein synthesis inhibition in Raji cells treated for 96 h with anti-CD20 or anti-CD22 ITs (black symbols) or the mixture of the respective unconjugated mAb and saporin-S6 (white symbols). Means ± S.D. of three independent experiments, each in triplicate, are given. SD never exceeded 10%.

To obtain the anti-CD20 and anti-CD22 ITs, saporin-S6 was conjugated to the anti-CD20 chimeric mAb rituximab and to the anti-CD22 murine mAb OM124, respectively, through the insertion of an artificial disulfide bond, as described in the methods section. The derivatization procedures with 2-iminothiolane allowed the insertion of an average of about three and two thiol groups per molecule for rituximab and OM124, respectively. In the case of saporin-S6, about one thiol group was inserted for each molecule (Table 1). After conjugation, the composition of the two purified ITs was analyzed by sodium dodecyl sulfate-polyacrylamide gel electrophoresis (SDS-PAGE) under non-reducing conditions. Densitometric analysis revealed RIP/mAb molar ratios of 1.85 and 1.41 for the anti-CD20 and anti-CD22 ITs, respectively (Figure 1). The inhibitory activity of the immunoconjugates on cell-free protein synthesis was evaluated in vitro using a rabbit reticulocyte lysate system and compared to saporin-S6 activity. The ability of saporin-S6 to inhibit cell-free protein synthesis was retained almost intact after derivatization and conjugation to both antibodies. A similar concentration causing 50% protein synthesis inhibition (IC_50_) was calculated for the two ITs (Figure 2a). The characteristics of the two immunoconjugates are summarized in Table 1 and Table 2.

### 2.2. Effect of the Immunotoxins on Raji Protein Synthesis and Cell Viability

The ability of the ITs to inhibit cellular protein synthesis was assayed in CD20-/CD22-positive Raji cells after 96 h of treatment (Figure 2b) and was compared to the mixture of unconjugated mAbs and saporin-S6. The anti-CD20 IT showed excellent efficacy with IC_50_ values of 1.99 nM, and it was able to almost completely abolish protein synthesis at a 10 nM concentration, expressed as RIP content. The anti-CD22 IT showed a higher inhibitory activity in Raji cells than the anti-CD20 IT with an IC_50_ of 0.060 nM and a complete protein synthesis inhibition already at a 1 nM concentration. The mixture of free RIP and either anti-CD20 or anti-CD22 mAbs produced scarce effect on Raji cell protein synthesis, with IC_50_ values >100 nM (highest tested concentration).

**Figure 3:**
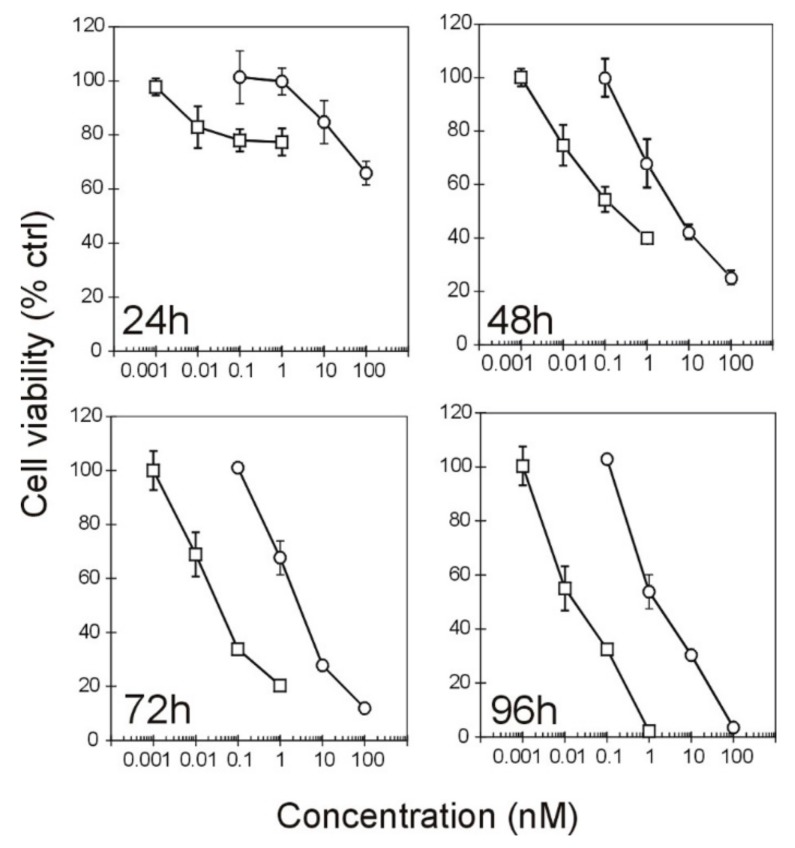
Effect of the immunotoxins on Raji cell viability. Raji cells were incubated for the indicated times with different doses of anti-CD20 IT (**○**) or anti-CD22 IT (**□**). Cell viability was measured by MTS salt reduction, as described in the methods section, and expressed as percentage of untreated cells. Means ± S.D. of three independent experiments, each in triplicate, are given. SD never exceeded 15%. At every tested time, the curves are significantly different from each other (*p* < 0.0001). MTS = 3-(4,5-dimethylthiazol-2-yl)-5-(3-carboxymethoxyphenyl)-2-(4-sulfophenyl)-2H-tetrazolium.

The viability of Raji cells after IT exposure was measured in dose-response experiments after different incubation times (24, 48, 72, and 96 h) to evaluate the minimum time required to observe a cytotoxic effect (Figure 3). The IT cytotoxicity is related to the incubation time. The dose-response curves showed similar tendencies in both cases. Even in this test, the anti-CD22 IT confirmed its two log higher toxicity than the anti-CD20. The maximum cytotoxic effect, corresponding to the complete abolishment of cell viability, was observed after 96 h only for the highest tested doses: 100 nM and 1 nM for anti-CD20 and anti-CD22 ITs, respectively. The mixture of free RIP and either anti-CD20 or anti-CD22 mAbs produced no relevant effect on Raji cell viability in the tested concentration range (data not shown). The concentrations causing 50% cell viability reduction (EC_50_) values for the anti-CD20 IT and anti-CD22 IT calculated after 96 h were 4.06 nM and 0.05 nM, respectively (Table 2). The effect of immunotoxins was also evaluated on viability of CD20-/CD22-negative Jurkat cells. For both immunotoxins, after 96 h of incubation, the EC_50_ calculated was higher than the maximum tested concentration (100 nM) (Table 2).

### 2.3. Evaluation of Internalization Time of the Immunotoxins

The binding of the ITs to the CD20 and CD22 membrane antigens in Raji cells was evaluated by cytofluorimetric analysis, after different incubation times with ITs. To allow binding and avoid the internalization of the complex, Raji cells were treated with ITs at a 10 nM concentration, for 30 min on ice. Cells were then incubated at 37 °C for different times ranging from 0 to 120 min. We considered as the maximum antigen binding the fluorescence intensity value obtained after 30 min incubation of cells with the ITs on ice, followed by 0 min exposure at 37 °C.

**Figure 4:**
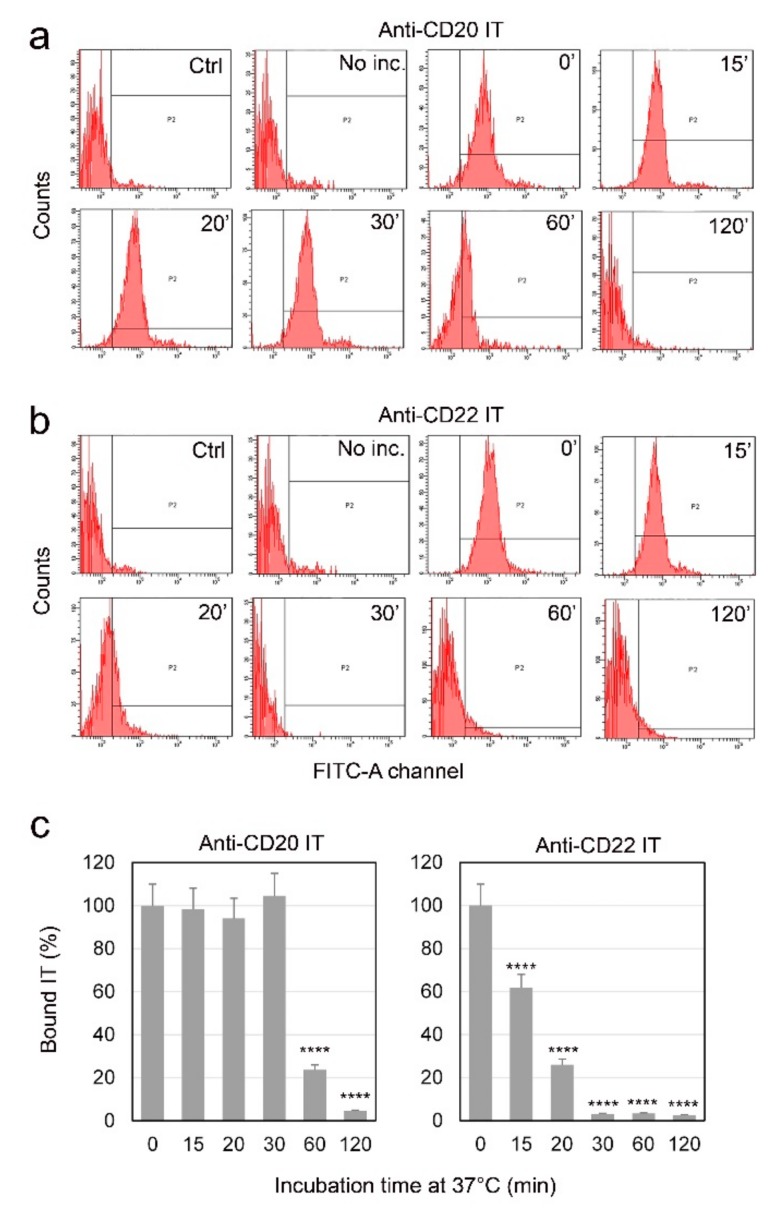
Evaluation of the internalization time of the antigen-immunotoxin complex by cytofluorimetric analysis in Raji cells. Samples were prepared by incubating cells with 10 nM anti-CD20 IT (**a**) or anti-CD22 IT (**b**) for 30 min on ice to allow the binding of the IT to the antigen, avoiding the internalization of the complex. After cell incubation for 0–120 min at 37 °C, the corresponding FITC-secondary antibody was added. Negative controls were carried out by incubating cells with complete medium alone (ctrl). A second series of controls were obtained without the 30 min pre-incubation at 0 °C and instead putting cells into contact with the IT for only an instant (No inc.). In Figure 4c, the percentage of cell membrane bound IT at the indicated times is reported. The bound IT is expressed as the percentage of mean fluorescence intensity values for each time point with respect to those of the 0 min samples, which was considered the maximum antigen binding. The values significantly lower than the 0 min samples are indicated by asterisks (**** *p* < 0.0001). The results are the means of three independent experiments.

The two ITs have a similar binding intensity to Raji cells at 0’ (compare histograms in Figure 4a,b 0’). In the case of the anti-CD20 IT (Figure 4a,c), the positivity to FITC remained unchanged from 0 to 30 min at 37 °C. The IT bound to the membrane significantly decreased after 60 min and was almost completely absent after 120 min, indicating the partial and complete internalization of the CD20-IT complex, respectively.

The anti-CD22 IT showed a faster internalization of the antigen-IT complex in comparison to the CD20 one (Figure 4b,c). In fact, after 15 min of incubation at 37 °C, the observed binding was already significantly lower than that observed for cells incubated for 0 min at 37 °C (*p* < 0.0001). After 20 min the IT bound to membrane resulted strongly decreased, and after 30 min, the complex was completely internalized.

### 2.4. Evaluation of Cell Death Pathways Induced by Immunotoxins in Raji Cells

**Figure 5:**
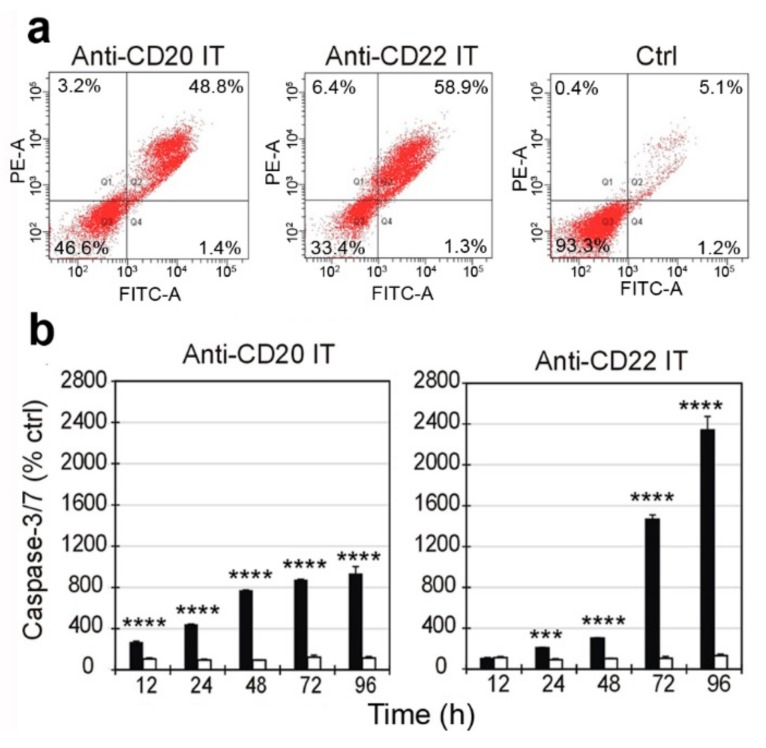
(**a**) Cytofluorimetric analysis of Annexin V/propidium iodide double staining of Raji cells treated for 96 h with 1 nM anti-CD20 IT or 0.01 nM anti-CD22 IT, i.e., the concentrations corresponding to their EC_50_ values. FITC-A channel (*x*-axis) is used for the detection of Annexin V-EGFP fluorescence. PE-A channel (*y*-axis) is used for the detection of propidium iodide fluorescence. (**b**) Caspase 3/7 activation in Raji cells exposed to 1 nM anti-CD20 IT or 0.01 nM anti-CD22 IT (black columns) or a mixture of unconjugated mAb and saporin-S6 (white columns). The expression of activated caspases was reported as percentage of untreated cell values. Means ± S.D. of three independent experiments, each in triplicate, are given. Statistical significance was determined by ANOVA/Bonferroni test. Asterisks indicate the significant difference in each experimental condition between IT and the mixture of mAb and RIP (**** *p* < 0.0001; *** *p* < 0.001).

The presence of membrane apoptotic and necrotic changes in Raji cells treated for 96 h with the ITs was evaluated by double staining with Annexin V-EGFP (AnnV) and propidium iodide (PI) at concentrations of 1 nM for anti-CD20 IT and 0.01 nM for anti-CD22 IT. As shown in Figure 5a, after exposure to ITs, approximately 50% (anti-CD20 IT) and 60% (anti-CD22 IT) of cells were positive for AnnV and PI double staining, indicating a late apoptosis stage. A very low percentage of necrotic cells (AnnV^−^/PI^+^) was evidenced for both ITs, 3.2% for anti-CD20 IT and 6.4% for anti-CD22 IT (Figure 5a), compared to approximately 0.5% in untreated cells.

The activation of effector caspases 3/7 was measured in Raji cells after 12, 24, 48, 72, and 96 h of treatment with the ITs at the same concentrations used for the AnnV/PI experiments. Both ITs caused a strong caspase 3/7 augmentation, but with different slopes of the activation curves. Raji cells treated with the anti-CD20 IT showed significant activation of caspase 3/7 already after 12 h (*p* < 0.0001). The caspase activation curve showed a fairly linear rise in the range of time tested, reaching approximately 900% of the value observed in untreated cells after 96 h (Figure 5b, left). In the case of the anti-CD22 IT, the caspase activation curve increased slowly over the first 48 h, becoming significant only after 24 h (*p* < 0.001). However, the caspases showed an exponential growth at 72 and 96 h, reaching approximately 2300% of untreated cells after 96 h (Figure 5b, right). In both cases, the mixture of unconjugated mAb and saporin-S6 at the same concentrations as the ITs produced no relevant activation of caspases 3/7 in Raji cells.

### 2.5. Evaluation of the Protective Effect of Apoptosis and Necroptosis Inhibitors Z-VAD and Necrostatin-1 and the H_2_O_2_ Scavenger Catalase on Raji Cells

**Figure 6:**
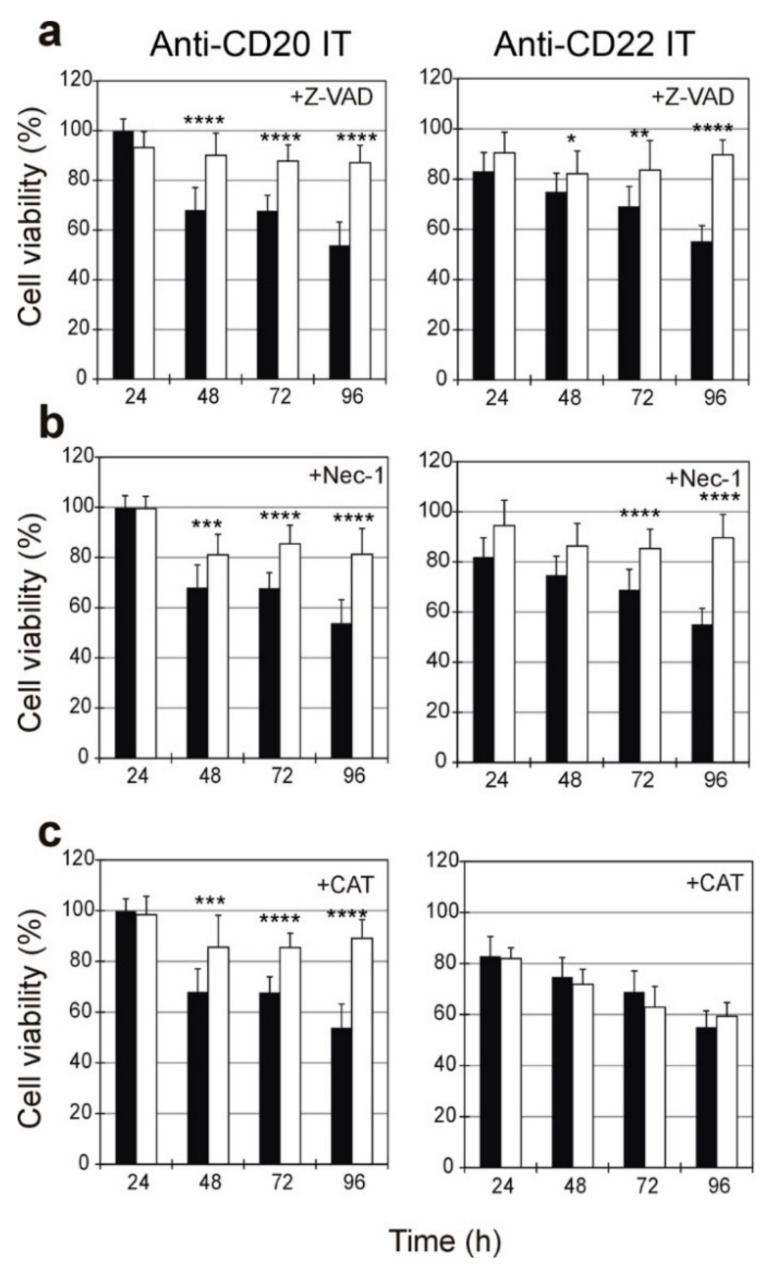
Viability of Raji cells treated for 24, 48, 72, and 96 h with 1 nM anti-CD20 IT (left) or 0.01 nM anti-CD22 IT (right) without (black columns) or in the presence (white columns) of 10 μM pan-caspase inhibitor (Z-VAD) (**a**), 10 μM necroptosis inhibitor necrostatin-1 (Nec-1) (**b**) or 10 U/mL hydrogen peroxide scavenger catalase (CAT) (**c**). Z-VAD, Nec-1 and CAT were added 3 h before the ITs, and the viability was measured after the indicated times. Means ± S.D. of at least three independent experiments, each in triplicate, are shown as the percentage of untreated cell values. Statistical significance was determined by ANOVA/Bonferroni test. Asterisks indicate the significant difference in each experimental condition between IT alone and IT plus inhibitors/scavenger (**** *p* < 0.0001; *** *p* < 0.001; ** *p* < 0.01; * *p* < 0.05).

**Figure 7:**
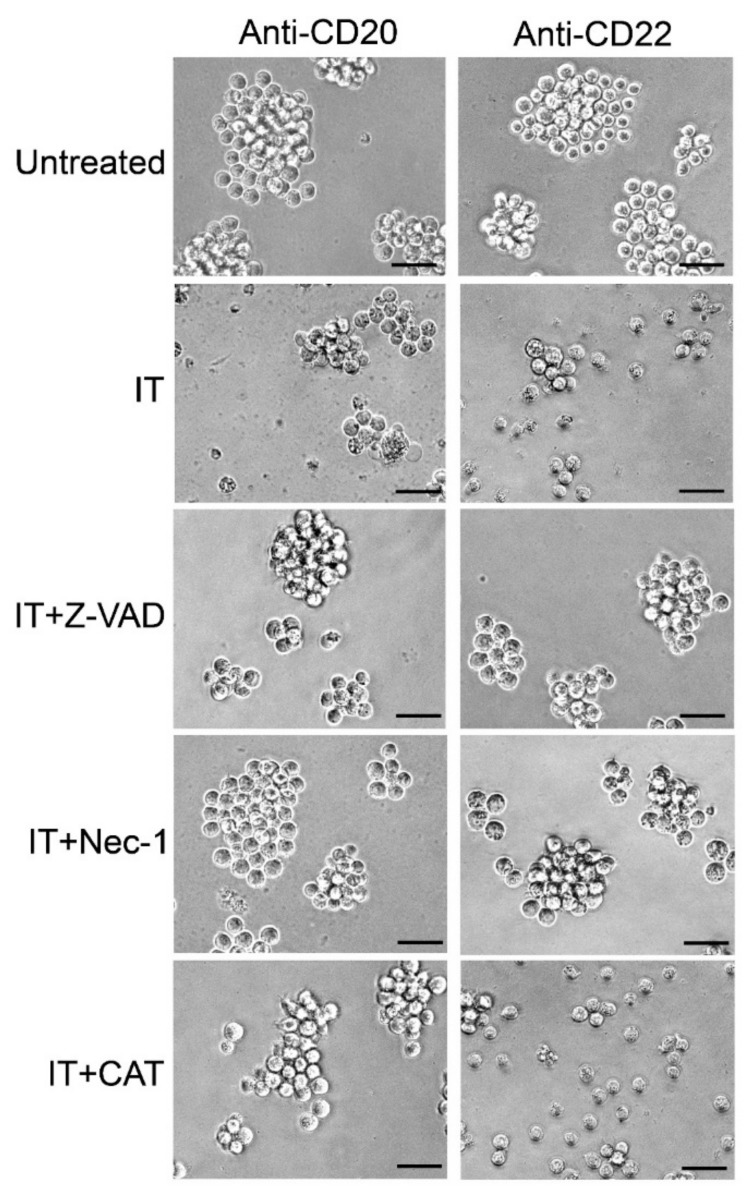
Morphological analysis of Raji cells assessed using phase-contrast microscopy. Cells were treated for 96 h with 1 nM anti-CD20 IT (left) or 0.01 nM anti-CD22 IT (right) alone (IT) or in the presence of 10 μM pan-caspase inhibitor (Z-VAD), 10 μM necroptosis inhibitor necrostatin-1 (Nec-1), or 10 U/mL hydrogen peroxide scavenger catalase (CAT). Z-VAD, Nec-1 and CAT were added 3 h before the ITs. Untreated cultures were grown in the absence of ITs. Magnification, 400 ×. Scale bars correspond to 50 µm.

To determine the role of apoptosis and necroptosis in IT-induced cell death, we designed experiments that included the pan-caspase inhibitor Z-VAD and the necroptosis inhibitor Nec-1. Additionally, the involvement of oxidative stress was investigated by including the reactive oxygen species (ROS) scavenger catalase. Raji cells were treated with the anti-CD20 IT and anti-CD22 IT at 1 nM and 0.01 nM concentrations, respectively. The viability was measured after different amounts of time, ranging from 24 to 96 h of exposure to ITs in the presence or absence of Z-VAD (10 µM), Nec-1 (10 µM), or catalase (10 U/mL), added 3 h before the IT treatment (Figure 6 and Figure 7).

The pan-caspase inhibitor Z-VAD was able to protect Raji cells from death triggered by ITs. As shown in Figure 6a, the protective effect of Z-VAD became significant from 48 h (*p* < 0.0001 for anti-CD20 IT, *p* < 0.05 for anti-CD22 IT) and increased over time, leading to a maximum protective effect on cell viability after 96 h. The necroptosis inhibitor Nec-1 protected Raji cells from IT-induced cell death similarly to Z-VAD (Figure 6b). The protective effect became significant from 48 h for the anti-CD20 IT (*p* < 0.001) and from 72 h for the anti-CD22 IT (*p* < 0.0001), leading to a maximum protective effect after 96 h. Surprisingly, different behavior was observed when cells were pretreated with catalase. In fact, catalase was able to protect cells from cytotoxicity induced by anti-CD20 IT with a similar trend than Z-VAD and Nec-1; its protective effect became significant after 48 h (*p* < 0.001) and reach its maximum at 96 h. By contrast, no protection was given by the scavenger in cells treated with anti-CD22 IT at any tested time (Figure 6c).

Visual inspection by phase-contrast microscopy of cells treated for 96 h with 1 nM anti-CD20 IT or with 0.01 nM anti-CD22 IT showed a strong cytotoxic effect with a marked reduction of cell viability compared to untreated cells (Figure 7). When pretreated with the inhibitors, Raji cells treated with the ITs appeared similar to untreated cells. Pretreatment with the scavenger catalase saved Raji cells from death triggered by anti-CD20 IT, but it was completely ineffective at rescuing cells treated with the anti-CD22 IT.

### 2.6. Combined Cytotoxic Effect of ITs with the Proteasome Inhibitors MG-132 and PS-341 and with the Chemotherapeutic Drug Fludarabine

**Figure 8:**
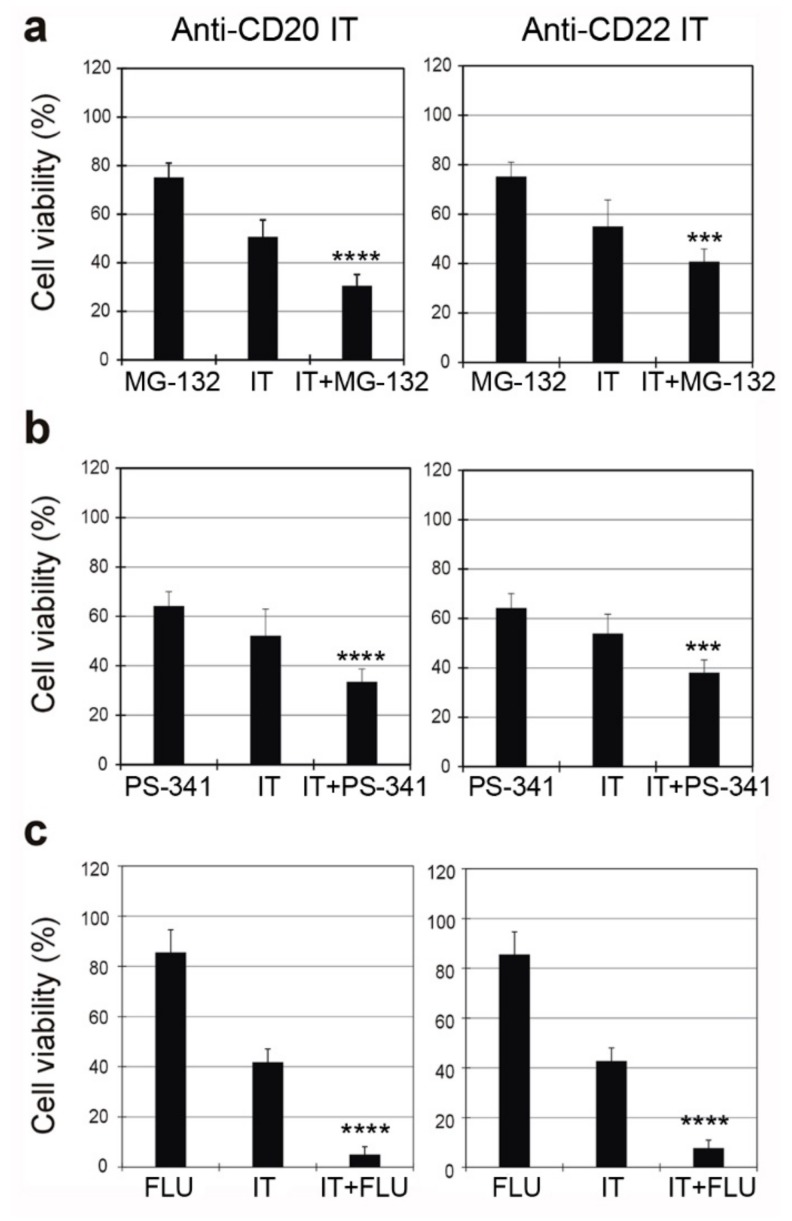
Viability of Raji cells treated for 96 h with 1 nM anti-CD20 IT (left) or 0.01 nM anti-CD22 IT (right) alone or in the presence of the proteasome inhibitors MG-132 (0.1 μM) (**a**) or PS-341 (1 nM) (**b**) or the purine analogue fludarabine (FLU) (0.75 μM) (**c**). Inhibitors and FLU were added 3 h before the ITs and maintained for the entire incubation time. Cell viability was determined after 96 h. Means ± S.D. of three independent experiments, each in triplicate, are showed as the percentage of the untreated cell values. Statistical significance was determined by ANOVA/Bonferroni test. Asterisks indicate the significant difference in each experimental condition between IT alone and IT plus the inhibitor or the purine analogue (**** *p* < 0.0001; *** *p* < 0.001).

To evaluate the possibility of enhancing the cytotoxic effect of the anti-CD20 and anti-CD22 ITs on Raji cells, we tested two proteasome inhibitors, MG-132 and PS-341 (bortezomib), giving them to Raji cells as single agents or in combination with the ITs (Figure 8a,b).

The sensitivity of Raji cells to both the anti-CD20 and anti-CD22 ITs was significantly augmented by co-incubation with MG-132. The combination of MG-132 and anti-CD20 IT produced a 2.8-fold enhanced toxicity compared to the proteasome inhibitor and a 1.4-fold enhancement compared to the IT (Figure 8a, left). The combination of MG-132 with the anti-CD22 IT gave similar results, showing a 2.4-fold increase in toxicity compared to MG-132 and a 1.3-fold increase compared to IT (Figure 8a, right). 

Similarly, responsiveness to the ITs was significantly augmented when cells were co-incubated with PS-341 (Figure 8b). The combination of PS-341 and the anti-CD20 IT produced a significant augmented effect in comparison to the single agents, showing a 1.7- and 1.3-fold enhanced toxicity compared to PS-341 and IT, respectively (Figure 8b, left). The combination of PS-341 with the anti-CD22 IT gave a significant 1.9- and 1.4-fold increase in toxicity compared to PS-341 and IT, respectively (Figure 8b, right).

The combination of the purine analogue fludarabine (FLU) and the anti-CD20 IT significantly reduced Raji cell viability compared to single compounds, resulting in a superadditive effect, showing a 6.6- and 2-fold enhanced toxicity compared to FLU and IT, respectively (Figure 8c, left). In combination with the anti-CD22 IT, FLU gave a 6.3- and 1.9-fold increase in cytotoxicity compared to FLU and IT, respectively (Figure 8c, right).

## 3. Discussion

The specific targeting of B-cells via restricted surface antigens has already been demonstrated to be an efficacious approach for immunotherapy against antigen-positive NHLs. In the last few years, several anti-CD20 and anti-CD22 ITs composed of mAbs linked to RIPs [15,16,29], bacterial toxins [30], ribonucleases [31], drugs [32,33], or radioisotopes [1,2] have demonstrated potent anti-tumor effects in vitro and in vivo in animal models and, in some cases, also in clinical trials.

The study of the IT mechanisms of action in target cells may help the design of new immunoconjugates with higher cytotoxic potential and specificity to target cells. In our study, we tested and compared in vitro the specific cytotoxic properties and the cell death pathways triggered by two ITs defined as anti-CD20 IT, which was obtained by the chemical coupling of saporin-S6 to the anti-CD20 mAb rituximab, and anti-CD22 IT, which was produced by coupling saporin-S6 to the anti-CD22 mAb OM124.

Despite CD20 and CD22 antigens being the most utilized targets for immunotoxin based NHL therapy, very little information is available about the pathogenic mechanism of cell intoxication.

Rituximab showed a 40% higher reactivity than OM124 for the cross-linking reagent 2-iminothiolane. As a consequence, a higher saporin-S6/antibody molar ratio was obtained for the anti-CD20 IT. After the conjugation processes to anti-CD20 and anti-CD22 mAbs, saporin-S6 retained almost the same enzymatic activity as native saporin-S6 on eukaryotic ribosomes (0.06–0.09 nM).

The ability of the ITs to kill lymphoma cells was tested in the CD20-/CD22-positive Raji cell line. In both cases after conjugation the cytotoxic activity of saporin-S6 and mAbs was significantly augmented. Despite the anti-CD20 IT showing a slightly higher saporin-S6 payload than the anti-CD22 IT, the latter resulted approximately in being 30-fold more efficient in cell protein synthesis inhibition and 80-fold more toxic to Raji cells. By analyzing the cytotoxicity curves at different time points, it is possible to see that the different behavior of the two ITs is maintained over time.

It is well known from the literature that the CD20 antigen is poorly internalized after ligand binding [34]. However, in previous papers we demonstrated that rituximab increases its internalization after conjugation with the plant toxin saporin-S6, giving an IT with a good specific cytotoxicity for lymphoma target cells [16,35]. The higher specific cytotoxic effect of the anti-CD22 IT could be expected on the basis of the more rapid CD22 internalization after ligand binding and its recycling to plasma membrane [36]. Actually, our experiments confirm the rapid internalization of the anti-CD22 IT, as the complex antigen/IT was completely internalized already after 30 min at 37 °C, while with the CD20/IT complex the same result was obtained after 120 min at 37 °C.

In agreement with the results reported for other saporin-S6-containing ITs [37,38,39], in the present study both the anti-CD20 IT and anti-CD22 IT were found to induce apoptosis in the target cells. However, differences in timing and intensity were observed in the activation of executioner caspases that were significantly augmented after 12 or 24 h of incubation with the anti-CD20 IT and anti-CD22 IT, respectively. The anti-CD22 IT caused a slightly delayed but exponential caspase activation that, after 72–96 h, reached much higher values than that caused by anti-CD20 IT. The retard in caspase activation observed for the anti-CD22 IT, in spite of its rapid internalization, could be justified by a different and slower routing of this IT with respect to the anti-CD20 IT. Finally, the anti-CD22 IT efficiently reaches its intracellular targets resulting in a stronger caspase activation, without ROS involvement, as commented below.

Despite the differences observed in caspase activation, the pretreatment with the pan-caspase inhibitor Z-VAD gave a good, even if incomplete, cell protection (approx. 90% survival) when used with each IT. This incomplete protection prompted us to evaluate other cell death mechanisms, taking into account that: (i) saporin-S6 and some other RIPs were reported to trigger multiple death pathways [25,40] and (ii) RIP conjugation to a specific carrier can modify its intracellular routing and consequently its toxicity pattern. 

Necroptosis is a recently identified programmed cell death, depending on the serine-threonine kinase receptor-interacting proteins 1 and 3. Nec-1 blocks necroptosis through the inhibition of serine-threonine kinase receptor-interacting protein 1 [41]. In our experiments, Nec-1 was able to protect Raji cells from the damage induced by the anti-CD20 and anti-CD22 ITs at a fairly similar level as Z-VAD, thus suggesting the involvement of both apoptosis and necroptosis, with a quite similar timing.

Oxidative stress was identified as being involved in cell death induced by a saporin-based conjugate directed against TfR1 [42]. Recently, it was reported that also the type 2 RIP stenodactylin is able to induce the early formation of ROS molecules in a neuroblastoma cell line and that several ROS scavengers can protect cells from RIP intoxication [40]. In our experiments, catalase significantly protected cells from damage induced by the anti-CD20 IT, but no protection was evidenced in cells treated with the anti-CD22 IT in the considered period of time. The cytotoxicity of anti-CD20 mAbs was previously demonstrated to be partially dependent on ROS generation in lymphoma cells [43,44]. Our data confirm the involvement of ROS in the cytotoxic effect of the anti-CD20 IT, but it appears to be absent for the anti-CD22 IT. This different behavior indicates that the induction of oxidative stress by ITs is not dependent on the toxic moiety but instead on the mAb/antigen interaction. The immunotoxin routing, depending mainly on the carrier moiety, can lead to RIP accumulation in specific compartments, such as the endoplasmic reticulum, where UPR can trigger ROS generation [45]. To date this is the only way to generate an oxidative stress in RIP intoxicated cells. The lack of oxidative stress in anti-CD22 IT treated cells suggests that massive endoplasmic reticulum accumulation and UPR are not involved in cell damage.

In vitro studies using rituximab-resistant cell lines showed that the development of rituximab resistance could be attributed to significant changes that occur in the CD20 antigen and in the deregulation of the ubiquitin-proteasome system [46,47]. Moreover, type 2 RIP cytotoxicity was shown to be partially reduced by proteasome activity [48], and bortezomib activity was enhanced by combination with an anti-CD22 mAb immunotherapy [49]. For these reasons, we were expecting a strongly augmented effect in the combined treatment with IT and a proteasome inhibitor. In our experiments, proteasome inhibition was able to increase cytotoxicity of both ITs, even if the combination gave a cytotoxicity lower than the sum of the single agents. The lack of superadditive effect suggests that our immunotoxins do not undergo a relevant cytoplasmic degradation.

We previously demonstrated that fludarabine strongly enhanced rituximab/saporin-S6 cytotoxicity [16]. The current experiments demonstrate a superadditive effect also when fludarabine was given in combination with the anti-CD22 IT. The ability of saporin-S6 to deadenylate poly(ADP-ribosyl)ated poly(ADP-ribose) polymerase in a cell free system was previously described [50]. If this occurs also in vivo, it would interfere with the DNA repair mechanism through the base excision repair complex inhibition, probably amplifying the effect of fludarabine. Altogether, these results indicate the advantage of a combination IT/FLU therapy with respect to proteasome inhibitor/IT therapy.

The biological properties of saporin-S6-containing ITs make them attractive molecules for the treatment of NHLs because their ability to induce cell death by more than one pathway makes the selection of tumor clones resistant to saporin-S6-induced cell death difficult.

Although some critical opinions about the clinical use of immunoconjugates that distrust their therapeutic potential have been expressed, the response rates observed in clinical trials have often been greater than those reported for conventional antiblastic drugs [8,51]. In past years, the clinical development of ITs has been hampered by several limitations, like immunogenicity and vascular leak syndrome. However, novel immunoconjugates aimed at reducing these side effects have been designed, and new and exciting opportunities for controlled drug delivery and release have been developed [52,53]. Furthermore, an anti-IL-2R IT was approved in 1999 by the U.S. Food and Drug Administration for the treatment of cutaneous T-cell lymphoma in adults [54]. Today, over thirty antibody-drug conjugates and ITs are in clinical trials, thus demonstrating that the magic bullet idea of Paul Ehrlich is still relevant [55]. The great interest in the field of institutional investigators and pharmaceutical companies is also suggested by the increasing number of patented immunoconjugates, and many researchers agree that immunoconjugates will likely become important actors in cancer therapy in the foreseeable future. The study of new ITs is today particularly interesting due to the availability of a new generation of mAbs, such as chimeric or humanized molecules, that are already at the clinical stage. The development of combination therapies may result in new effective options for the treatment of cancer, and the knowledge of the mechanism of action of the substances is mandatory in order to design proper effective protocols.

## 4. Materials and Methods

### 4.1. Immunotoxins

Saporin-S6 was purified from the seeds of *Saponaria officinalis* [19]. Anti-CD20 rituximab/saporin-S6 IT and anti-CD22 OM124/saporin-S6 IT were produced as described in [15,16]. Briefly, mAbs and saporin-S6 were dissolved in 50 mM sodium borate buffer, pH 9.0 and were derivatized by adding 2-iminothiolane (Sigma-Aldrich, St. Louis, MO, USA), as described in [56]. MAbs and the reduced RIP were allowed to react for 16 h at room temperature. The resulting conjugates were separated from RIP homopolymers and free antibody by gel filtration on a Sephacryl S-200 high-resolution column (100 cm × 2.5 cm) (GE-Healthcare, Buckinghamshire, UK), equilibrated, and eluted with phosphate-buffered saline (PBS, 0.14 M sodium chloride in 5 mM sodium phosphate buffer, pH 7.4).

The immunoconjugates were analyzed under non-reducing conditions by SDS-PAGE on a 4–15% PhastGel gradient, and then stained with Coomassie brilliant blue and analyzed, as described in [16]. Molecular weight markers were from Sigma: myosin (205 kDa), b-galactosidase (116 kDa), phosphorylase B (97 kDa), bovine serum albumin (66 kDa), ovalbumin (45 kDa), carbonic anhydrase (29 kDa).

### 4.2. Cells

The activity of the conjugates was assayed in the CD20-/CD22-positive Raji cell line (ATCC number CCL-86™) and in the non target Jurkat cell line (ATCC number TIB-152™). Cells were from long term culture in our department. The cells were cultured in RPMI 1640 medium supplemented with 10% heat-inactivated fetal bovine serum (FBS), 2 mM L-glutamine, 100 U/mL penicillin and 100 µg/mL streptomycin (hereafter named complete medium), cultured at 37 °C in a humidified environment with 5% CO_2_ in a HeraCell Haereus incubator (Hanau, Germany) and routinely checked for the absence of Mycoplasma infection. The viability was checked before each experiment by Trypan blue (BioWhittaker, Vervies, Belgium) dye exclusion. Flasks and plates were from Falcon (Franklin Lakes, NJ, USA). All the other cell culture reagents were from Sigma-Aldrich.

### 4.3. Reagents and Kits

Caspase activity was evaluated using the luminescent kit Caspase-Glo™3/7 Assay (Promega Corporation, Fitchburg, Wisconsin, USA). Morphological membrane changes were detected using Annexin V-EGFP/PI detection kit (Biovision, Mt. View, CA, USA). Viability was measured using the colorimetric CellTiter 96^®^ Aqueous One Solution Cell Proliferation Assay (Promega). The CellTiter 96^®^ Aqueous One Solution Reagent contains the tetrazolium compound 3-(4,5-dimethylthiazol-2-yl)-5-(3-carboxymethoxyphenyl)-2-(4-sulfophenyl)-2H-tetrazolium (MTS), and an electron coupling reagent (1-methoxy phenazine methosulfate—PMS). The liquid scintillation cocktail was the Ready-Gel (Beckman Instrument, Fullerton, CA, USA). The proteasome inhibitors PS-341 and MG-132, the necroptosis inhibitor Necrostatin-1 and the pan-caspase inhibitor carbobenzoxy-valyl-alanyl-aspartyl-[O-methyl]fluoromethylketone (Z-VAD-fmk) were supplied by Vinci-Biochem (Florence, Italy). The hydrogen peroxide scavenger catalase (CAT) and fludarabine (FLU) were purchased by Sigma-Aldrich. For SDS-PAGE, precasted gels and buffer strips obtained from GE Healthcare were used. Other reagents used were from Merck (Darmstadt, Germany), Carlo Erba (Milano, Italy) and Sigma-Aldrich.

### 4.4. Cell Protein Synthesis Inhibition Assays

The inhibitory activity of IT and free RIP on protein synthesis was evaluated using a cell-free rabbit reticulocyte lysate system. After reduction with 20 mM 2-mercaptoethanol for 30 min at 37 °C, the IT and RIP were diluted and added to the reaction mixture as previously described [57]. Each experiment was carried out in duplicate. The concentration of IT, expressed as RIP content, causing 50% inhibition of [4,5-^3^H]leucine incorporation (IC_50_) was calculated by linear regression analysis.

The inhibitory activity of free RIP and ITs was also evaluated on Raji cells as described in [37]. Cells (4 × 10^4^/well) were seeded in 96-well microtiter plates in 100 µL of complete medium in the presence of 100 µL of IT added at final concentrations ranging from 0.01 to 100 nM. Control samples were run with RIP alone, mAb alone, or a mixture of unconjugated anti-CD20 or anti-CD22 mAb and RIP. After 96 h, 1 µCi of L-[4,5-^3^H]leucine was added to each well. After further 6 h, the cells were harvested with an automatic cell harvester (Skatron Instruments, Lier, Norway) onto glass-fiber diskettes. Cell-incorporated radioactivity was determined by a β-counter (Beckman Coulter, Brea, CA, USA) with Ready-Gel scintillation liquid containing 0.7% acetic acid. The IC_50_ values were calculated by regression analysis.

### 4.5. Cell Viability Assay

Cell viability was evaluated using the colorimetric CellTiter 96^®^ Aqueous One Solution Cell Proliferation assay. Cells (2 × 10^4^/well) were seeded in 96-well microtiter plates in 100 µL of complete medium. After 24 h, the cells were incubated in the absence or in the presence of ITs or the mixture of unconjugated RIP and mAb at the desired concentrations in complete medium. After the indicated times, 20 µL/well of kit solution was added. After 1 h of incubation at 37 °C, the absorbance at 492 nm was measured by a microtiter plate reader Multiskan EX (Thermo Labsystems, Helsinki, Finland). Cell viability was also evaluated on non-target Jurkat cell line incubated with the immunotoxins for 96 h at the same conditions above described.

Experiments with cell death inhibitors and CAT were carried out by pretreating cells with 10 μM Z-VAD, 10 μM Nec-1, or 10 U/mL CAT (the highest concentrations found to be non-toxic to Raji cells in preliminary tests). Experiments with proteasome inhibitors and FLU were carried out by pretreating cells with 0.1 μM MG-132, 1 nM PS-341, or 0.75 µM FLU (the highest concentrations giving a toxicity for Raji cells between 15% and 35% in preliminary tests). All the above reported reagents were added to cells 3 h before the treatment with the ITs and maintained for the entire incubation times.

### 4.6. Cell Binding Assay

The time required for the antigen-IT complex to be internalized into Raji cells was evaluated by flow cytometry. Cells (5 × 10^5^) were incubated in cytometer tubes in a volume of 200 µL with anti-CD20 and anti-CD22 ITs at a final concentration of 10 nM for 30 min at 0 °C to allow the binding of the IT to the antigen and avoid the internalization of the antigen/IT complex. Afterwards, the samples were kept at 37 °C for different times (0, 15, 20, 30, 60, and 120 min). Negative controls were carried out by incubating cells with complete medium alone. Other controls were not pre-incubated for 30 min at 0 °C with the IT but instead exposed to the IT only for a very short contact time of a few seconds (No inc.). After the incubation time, the cells were washed twice in PBS containing 1% FBS, and incubated again for 30 min on ice in a volume of 50 µL/vial containing 0.1 µL of anti-mouse-FITC (for the anti-CD22 IT) or 0.1 µL of anti-mouse-FITC + 0.1 µL IgG anti-human-FITC (for the anti-CD20 IT). After three washes as above, the samples were fixed with PBS containing 70% ethanol. Cells were analyzed by flow cytometry on a FACSAria BD analyzer using FACSDiva software (Franklin Lakes, NJ, USA). The amount of bound IT was expressed as the percentage of MFI values for each time point with respect to that of samples incubated for 0 min at 37 °C, which was considered as the highest value because in this case binding is not followed by internalization.

### 4.7. Evaluation of Apoptosis

Apoptotic cell death was assessed using a flow cytometry Annexin V-EGFP/PI detection kit and by a luminescent reagent detecting caspase activity. Before flow cytometry, cells (2 × 10^5^/ 1 mL complete medium) were seeded in a 24-well microtiter plate, and after incubation with the ITs or unconjugated RIP + mAb, the cells were pelleted at 400 × *g* for 5 min, washed in 2 mL of complete medium, pelleted, and resuspended in 294 µL of binding buffer. Annexin V-EGFP (3 µL) and PI (3 µL) were added. After 10 min incubation in the dark at room temperature, cells were analyzed by flow cytometry. Data were analyzed as above described. The caspase-3/7 activity was assessed by the luminescent Caspase-Glo™3/7 Assay as described in [39]. Briefly, cells (2 × 10^4^/well) were seeded in 96-well microtiter plates in 40 µL of complete medium. Cells were treated with 40 µL of complete medium containing the ITs or unconjugated mAb and saporin-S6 to reach the desired concentration. After incubation for the indicated amounts of time, 80 µL/well of caspase kit reagent was added. The plates were shaken at 420 rpm for 1 min and then incubated for 20 min at room temperature in the dark. The luminescence was acquired (integration time 10 s) by a Fluoroskan Ascent FL (Thermo Labsystems) and values were normalized to cell viability.

The morphological analysis of the treated cells was conducted through phase contrast microscopy, directly in 96-well plate, using an inverted microscope Nikon Eclipse TS100 (Nikon, Melville, NY, USA), at the end of the above described experiments.

### 4.8. Statistical Analyses

Statistical analyses were conducted using XLSTAT-Pro software, version 6.1.9, 2003 (Addinsoft, Inc., Brooklyn, NY, USA). The results are presented as the means ± S.D. of three different experiments. The data were analyzed using ANOVA/Bonferroni test. The Dunnett’s test was used in addiction to ANOVA, when necessary.

## Figures and Tables

**Table 1:** Characteristics of the anti-CD20 IT and anti-CD22 IT.

Immunotoxin	Linker ^#^/Ab	Linker ^#^/RIP	RIP/Ab
anti-CD20 IT	(mol/mol)	(mol/mol)	(mol/mol)
	3.32	0.92	1.85
anti-CD22 IT	2.31	1.09	1.41

^#^ The ribosome-inactivating protein (RIP) and the mAbs were modified using 2-iminothiolane linker, and the number of SH groups inserted per molecule is reported.

**Table 2:** Effect of the anti-CD20 IT and anti-CD22 IT on cell-free and cellular systems.

Immunotoxin	Cell-Free	Raji Cells ^§^		Jurkat Cells ^#^
	IC_50_ * (nM)	IC_50_ * (nM)	EC_50_ * (nM)	EC_50_ * (nM)
anti-CD20 IT	0.08	1.99	4.06	>100
anti-CD22 IT	0.09	0.06	0.05	>100

* The IC_50_ and EC_50_ of the immunotoxins refer to the RIP content. IC_50_, concentration causing 50% protein synthesis inhibition; EC_50_, concentration causing 50% cell viability reduction. ^§^ The IC_50_ and EC_50_ on Raji cells were calculated after 96 h of incubation with the immunotoxins. **^#^** The EC_50_ on Jurkat non target cells (CD20-/CD22-negative) was calculated after 96 h of incubation with the immunotoxins.
